# Development of a Positive Body Image Chatbot (KIT) With Young People and Parents/Carers: Qualitative Focus Group Study

**DOI:** 10.2196/27807

**Published:** 2021-06-16

**Authors:** Francesca Beilharz, Suku Sukunesan, Susan L Rossell, Jayashri Kulkarni, Gemma Sharp

**Affiliations:** 1 Monash Alfred Psychiatry Research Centre Monash University Melbourne Australia; 2 Swinburne Business School Swinburne University of Technology Melbourne Australia; 3 Centre for Mental Health Swinburne University of Technology Melbourne Australia; 4 Psychiatry St Vincent's Hospital Melbourne Australia

**Keywords:** body image, eating disorder, chatbot, conversational agent, artificial intelligence, mental health, digital health, design

## Abstract

**Background:**

Body image and eating disorders represent a significant public health concern; however, many affected individuals never access appropriate treatment. Conversational agents or chatbots reflect a unique opportunity to target those affected online by providing psychoeducation and coping skills, thus filling the gap in service provision.

**Objective:**

A world-first body image chatbot called “KIT” was designed. The aim of this study was to assess preliminary acceptability and feasibility via the collection of qualitative feedback from young people and parents/carers regarding the content, structure, and design of the chatbot, in accordance with an agile methodology strategy. The chatbot was developed in collaboration with Australia’s national eating disorder support organization, the Butterfly Foundation.

**Methods:**

A conversation decision tree was designed that offered psychoeducational information on body image and eating disorders, as well as evidence-based coping strategies. A version of KIT was built as a research prototype to deliver these conversations. Six focus groups were conducted using online semistructured interviews to seek feedback on the KIT prototype. This included four groups of people seeking help for themselves (n=17; age 13-18 years) and two groups of parents/carers (n=8; age 46-57 years). Participants provided feedback on the cartoon chatbot character design, as well as the content, structure, and design of the chatbot webchat.

**Results:**

Thematic analyses identified the following three main themes from the six focus groups: (1) chatbot character and design, (2) content presentation, and (3) flow. Overall, the participants provided positive feedback regarding KIT, with both young people and parents/carers generally providing similar reflections. The participants approved of KIT’s character and engagement. Specific suggestions were made regarding the brevity and tone to increase KIT’s interactivity.

**Conclusions:**

Focus groups provided overall positive qualitative feedback regarding the content, structure, and design of the body image chatbot. Incorporating the feedback of lived experience from both individuals and parents/carers allowed the refinement of KIT in the development phase as per an iterative agile methodology. Further research is required to evaluate KIT’s efficacy.

## Introduction

### Background

Mental disorders are a major public health concern, with a 29% lifetime prevalence rate across the general population [1]. In addition to reduced quality of life and individual impact, mental disorders have a significant global economic cost, which has been predicted at US $16.3 trillion worldwide from 2011 to 2030 [2]. Eating disorders have the highest mortality rate of any mental health diagnosis [3,4], and global prevalence rates have doubled from 3.4% to 7.8% of the population from 2000 to 2018 [5]. Despite the significant physical, mental, and social consequences associated with eating disorders, more than 75% of these individuals do not access appropriate treatment [6].

A major risk factor for developing eating disorders or other serious mental health conditions is body image dissatisfaction or concern [7]. This risk is increased by using social media, particularly image-based platforms such as Facebook, Instagram, and TikTok [8]. For example, using social media for 30 minutes a day, particularly looking at photos of peers and celebrities, has been shown to negatively impact women’s body image and mood [9]. Over 3.6 billion people worldwide used social media in 2020, with many young people spending a significantly increasing amount of time on these platforms [10]. Consequently, social media, where young people are spending their time, presents a unique pathway to prevent and intervene with body image and eating concerns.

Guidelines for the evidence-based treatment of eating disorders recommend prevention and early intervention strategies for the best prognosis, with most individuals developing these concerns as young people [7,11]. Therefore, there is a need for early intervention and support delivery that is accessible and available to young people. The therapeutic approach with the strongest evidence base across the full spectrum of eating disorder presentations is enhanced cognitive behavioral therapy (CBT-E) where the core psychopathology addressed in treatment is the overevaluation of weight and shape (broadly, negative body image) [12]. Psychoeducation, or communicating relevant information about mental health conditions and treatments, is deemed an essential part of all psychological treatments, including CBT-E [12,13], especially given the wealth of misinformation in the general community about “healthy” eating and weight [11]. Psychoeducation is associated with small yet significant improvements in body image, which is further enhanced when combined with additional interventions [14,15].

The development of alternative coping skills is another requirement of evidence-based treatments [16], such as mindfulness practice, which involves the nonjudgemental awareness and acceptance of the present moment. Mindfulness training has been shown to significantly improve body image dissatisfaction and can effectively be delivered online as brief microinterventions [17-19]. When combined with psychoeducation, these brief evidence-based interventions (microinterventions) are likely to be beneficial in reducing body image concerns. Therefore, providing an accessible platform where individuals and support people can access evidence-based psychoeducation and coping skills may assist in addressing negative body image, and, in turn, potentially intervene in the development of eating disorders.

### Prior Work

Digital mental health interventions have been increasing in popularity and efficacy, with chatbots being one such format. A chatbot allows the provision of information and services through a message-based interface, which is available on messaging platforms, websites, and apps. The conversational language and format of chatbots are relevant in connecting with youth, and are a promising avenue to supplement traditional support services [20]. Chatbots in the mental health sphere offer several benefits, including wider accessibility, instantaneous responses, and low or no cost to users, and can act as a stepping stone to link with more individualized or alternative services [21]. An additional barrier to accessing traditional services can be mental health stigma or lack of available services, where individuals may feel uncomfortable or unable to reach out for help. Digital mental health services can help bridge this gap, as anonymously conversing with artificial intelligence can feel less intimidating, particularly for young people who have grown up in the digital age [21].

The evidence for chatbots as supplements to mental health support is growing, with generally positive outcomes [20,22]. A recent systematic review and meta-analysis of 12 studies suggested that while heterogeneity among chatbot studies limits the interpretations of pooled efficacy, individual studies have evidenced improvements in symptoms of depression, anxiety, and general coping skills [23]. Owing to the limited conclusions that can be made, the authors recommend that chatbots be used to supplement treatment from mental health professionals rather than as a replacement. A scoping review of 41 chatbots reported that the most common uses of chatbots are therapy provision, information delivery, and screening, with the majority focused on mood and anxiety disorders [24]. However, to the authors’ knowledge, there are no chatbots currently available that specifically target the increasingly common issue of negative body image. Given the rise of social media leading to increased appearance comparisons, “proeating disorder” content online, and “health” misinformation, there is a need for a reliable, evidence-based, and supportive digital mental health service within the field so users can receive more timely support for body image concerns.

### Our Study

Based on this gap in service provision, we initiated the development of a novel body image chatbot. The chatbot provides psychoeducation and coping skills targeting body image concerns, for the purposes of prevention and supplementing traditional forms of treatment. This study aimed to assess the preliminary acceptability and feasibility of such a chatbot by collecting qualitative data from focus groups. This was an early stage evaluation, predominantly focused on the content, structure, and design of the chatbot in order to refine the design prior to launch, and the assessment of efficacy.

## Methods

### KIT Intervention

The chatbot, called “KIT,” is a conversational agent designed to support people with concerns surrounding body image and eating issues, as well as their loved ones (eg, parents, partners, and friends). The preliminary conversational content and decision tree for KIT was developed by the authors, in collaboration with the helpline and communications teams at the Butterfly Foundation (a national charity based in Australia, which provides support for body image and eating disorders). The conversation content was based on evidence-based information/interventions for eating disorders, specifically, psychoeducation, cognitive behavioral therapy (CBT), acceptance commitment therapy (ACT), and mindfulness [25,26], and adapted for delivery by a chatbot. Owing to the short and simple style of conversations KIT was designed to deliver, we were highly selective in the therapeutic elements we chose from CBT (eg, education on cognitive distortions or unhelpful thinking styles), ACT (eg, practicing detaching from unhelpful thoughts via cognitive defusion exercises), and mindfulness (eg, mindful breathing) [17,25,26].

In an effort to enhance the co-design of KIT, preliminary online forums were held with young people aged 13 to 18 years who self-identified as having lived experience with body image concerns and/or eating disorders. Please note that the reason this age range was chosen was to ensure that KIT’s dialog was understandable to the youngest users possible, not that KIT’s usage is limited to people aged 13 to 18 years. We also ran preliminary online forums with parents/carers of people with lived experience of body image concerns and/or eating disorders. The preliminary forums were crucial to assist with the refinement of the dialog, particularly devising shorter and more conversational ways of delivering educational information, as well as the design of the decision tree. The importance of involving end users in a software project has garnered strong support over recent years.

A professional chatbot production company, Proxima, using Iris Conversational Intelligence software [27], built the prototype chatbot based on the co-designed decision tree and conversation content. The prototype chatbot took the form of a webchat and was accessible on both mobile and desktop devices.

**Figure 1:**
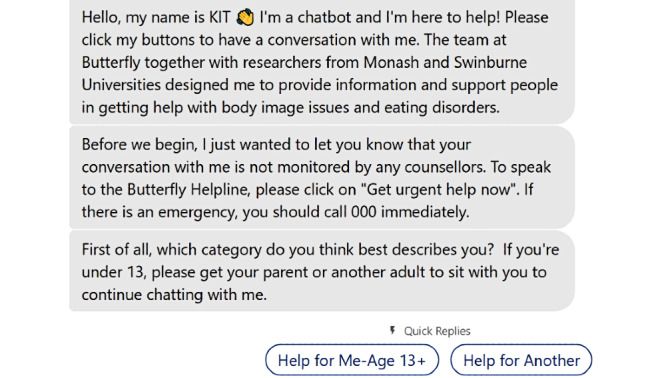
KIT’s welcome message for users explaining capabilities and prompts for users to choose the “help for me” or “help for another” pathway.

In terms of the specific chatbot content, each interaction with KIT began with a welcome message explaining the purpose and capabilities of the chatbot (Figure 1). This included directions to proceed through KIT by selecting buttons and information that the service was not monitored by any counsellors. Users were directed to speak to the Butterfly helpline or call emergency services if they required a higher level of assistance. KIT then asked the primary reason the user was there (“help for me [aged 13+]” or “help for another”). Once users were directed down one of these two pathways, they could self-direct to various psychoeducation or microintervention contents. The minimum age to use KIT was determined to be 13 years, as this is generally the age at which young people are permitted to sign up for a social media account [28].

**Figure 2:**
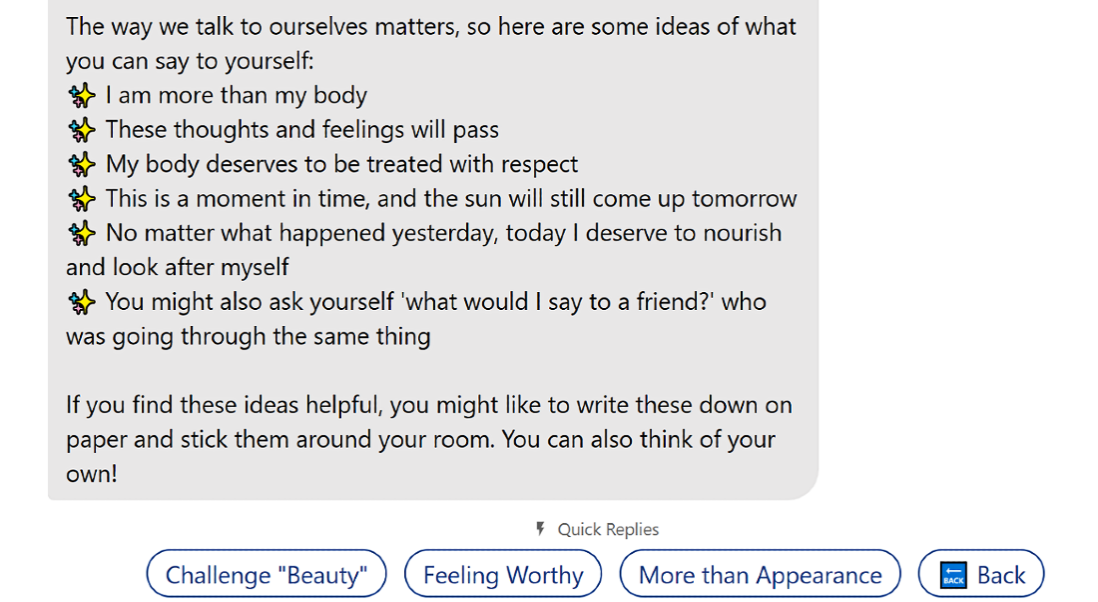
An example of one of KIT’s coping skills conversations, “Positive Self-Talk,” with prompts to continue the conversation with other skills that also aim to promote positive body image.

For people aged 13 years or above, KIT included psychoeducation conversations regarding body image, eating disorders and body dysmorphic disorders, health impacts, risk and protective factors, how to seek help, and support during the COVID-19 pandemic. The coping skills section covered strategies for managing social media, “busting myths” about food, comparisons and self-perception, challenging beauty ideals, mindfulness, managing difficult thoughts and emotions, body positive self-talk, enjoyable movement, and improving self-worth (Figure 2).

**Figure 3:**
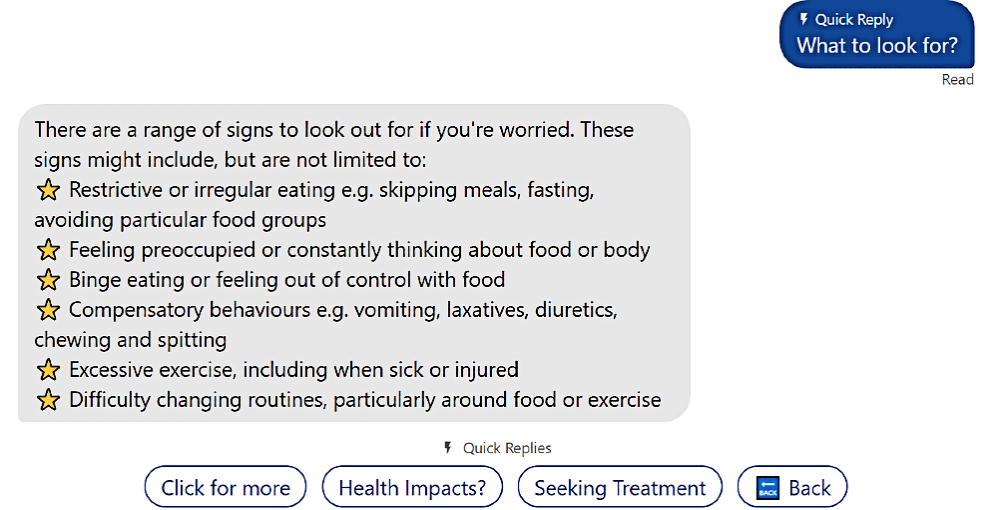
An example of one of KIT’s psychoeducation conversations in the “help for another” pathway where the user has asked “What to look out for?” as in eating disorder warning signs. The “Click for more” option provides further warning signs.

The pathway for people seeking to help someone else explored similar psychoeducation conversations, with the addition of further information regarding warning signs and treatment pathways (Figure 3). An additional section was included where users could seek support for themselves, including individual support and information about support groups. For all users, KIT included a “get urgent help now” section that was linked to the Butterfly helpline and other crisis support services.

**Figure 4:**
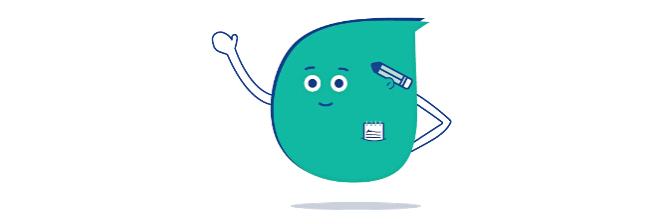
The main pose for the chatbot character of KIT.

To increase the appeal of the chatbot KIT service, a chatbot “character” (Figure 4) was designed by a professional graphic design company, Yoke, in collaboration with the authors and the helpline and communications teams at the Butterfly Foundation. Preliminary online forums were held with young people and parents/carers to inform the feature development of the character prior to the design by the graphic design company.

### Participants

Online advertisements were placed on Butterfly Foundation social media pages for people living in Australia (aged 13 to 18 years) with lived experience of body image concerns and/or eating disorders, and parents/carers of people with these lived experiences. Potential participants completed an online expression of interest form linked to the advertisement and were then invited (by the author GS or FB) to participate in the online focus group based on availability.

There were two focus groups for parents/carers, which included participants aged 46 to 57 years, with two males and six females, evenly split into groups of four. There were four focus groups for participants with lived experience (aged 13-18 years) seeking help for themselves. In each of these groups, there were four to five participants, with a total of 17 participants. Among these participants, there were five males, 10 females, one gender diverse individual, and one transgender individual. All participants were reimbursed with an Aus $30 (approximately US $23) online gift voucher for their focus group participation.

### Data Collection

Semistructured interview questions were used to guide the focus group discussion based on the capacity to gather in-depth descriptive data. The interview guide was developed by the research team. Example questions included “How likely would you be to recommend KIT to someone else?” and “How easily were you able to find the information you were looking for?”. The questions focused on the content, structure, and design of the chatbot, as well as the design of the chatbot character. The use of a semistructured interview allowed the researchers to explore the research questions, while permitting for flexible responses and any follow-up questions.

The focus groups were conducted by the authors GS and FB, who both have doctoral-level training in psychology and practice as clinical psychologists. All focus groups were conducted online via Zoom, over a period of 2 weeks in October 2020. Focus groups lasted from 61 to 86 minutes, with an average of 75 minutes.

### Data Analysis

Qualitative data analysis was conducted using thematic analysis, following the guidelines of Braun and Clarke [29]. The audio recordings for all focus groups were transcribed by a professional transcription service, which were read thoroughly multiple times. The data were coded by the author FB based on basic-level content and then grouped into appropriate categories. The themes were developed to capture the key aspects of participants’ responses, which were regularly reviewed by GS.

### Ethics

This project was approved by the Monash University Human Research Ethics Committee (MUHREC ID: 22527). All participants provided either written or audio-recorded verbal consent to participate in the focus group and the audio recording of the focus group, with parental/guardian consent recorded for all participants under the age of 18 years.

## Results

The results of the qualitative analysis are presented together for both types of focus groups as the overarching themes were identical (chatbot character and design, content presentation, and flow). Quotes for each of the subthemes are presented in Table 1.

**Table 1:** Themes, subthemes, and quotes from the focus groups.

Themes and subthemes			Quote number		Data extraction
**Key theme 1: Chatbot character and design**					
	Diverse appeal	1		*To me, KIT felt like non-binary, and I know it’s like a ridiculous thing, but I felt seen by this little character drawing, and I think that that never happens, so I really appreciate that, especially because gender diverse folks and eating disorders, there’s a really high correlation of the two.* [age 17 years, gender diverse, “help for me” group]	
	Diverse appeal	2		*I like how it’s very gender non-specific. It doesn’t scream like either male or female or anything like that, it’s just very basic, very good, so that appeals to everyone.* [age 17 years, transgender male, “help for me” group]	
	Diverse appeal	3		*I also like how KIT doesn’t have like really a body as much? I think that’s a good thing to avoid, triggers and getting to that minefield.* [age 18 years, male, “help for me” group]	
	Diverse appeal	4		*I love the fact that KIT is essentially just a head with arms coming out so we’ve taken away any sense that it could be a body and I think that’s really important.* [age 51 years, male, “parent/carer” group]	
	Diverse appeal	5		*I like its expressions and I love that you’ve incorporated all those in non-gender way, it’s fantastic.* [age 53 years, female, “parent/carer” group]	
	Visual appeal	6		*I think like helpful, problem-solving, like that second expression with the light-bulb, it’s like “let’s figure something out here.” I was going to say helpful, curious, that kind of thing.* [age 18 years, male, “help for me” group]	
	Visual appeal	7		*I think he looks really kind of positive and cheerful. I think automatically, all of his expressions look really happy would come across to me.* [age 15 years, female, “help for me” group]	
	Visual appeal	8		*Ten out of ten! Wholesome, very good looking bot. I love it!* [age 17 years, female, “help for me” group]	
	Visual appeal	9		*I think it’s like kind and simple? Like it’s uncomplicated and I think that’s a really positive thing because often when you’re looking for information, it’s really full-on and whereas this just looks like “oh this is going to be simple.”* [age 53 years, female, “parent/carer” group]	
	Visual appeal	10		*It’s got a cheery disposition so you know, it probably encourages you to at least investigate what’s on offer and if you’re feeling down or lost, hopefully you can get something positive out of.* [age 54 years, female, “parent/carer” group]	
**Key theme 2: Content presentation**					
	Brevity	11		*I feel like there’s a lot of information in there and it’s all really good information, but it’s all kind of piled onto you, and that can be quite overwhelming for some people.* [age 15 years, female, “help for me” group]	
	Brevity	12		*I definitely think for a chatbot, you want it to be little text things, not full chunks so I think all the information and the content was really good but if you did split it up into smaller bits, that you could click “more” if you wanted to see more. It would make it seem more like an interactive chat.* [age 15 years, female, “help for me” group]	
	Brevity	13		*Because it’s in big chunks of text, it sometimes gets a bit hard to read and I liked the ones where it had emoji dot points.* [age 17 years, transgender male, “help for me” group]	
	Brevity	14		*I love the way that the information is simplified and then there’s the link that goes to the detailed pages, because I remember when you’re in that initial teary “mum phase,” when you were going straight to those pages, it was just “Whoa!” whereas this is “oh, go here for the more information” and that gives your brain the time to adjust and be ready so I loved that about it.* [age 53 years, female, “parent/carer” group]	
	Brevity	15		*I think it’s important to have the links to allow people to go and look for more information for when you’re at that point and ready to seek more, but I think what everyone’s saying about the need for it to be more punchy in the speech bubble is probably the key thing. You must want the facts I guess, but you also need to be able to look further if you want additional information.* [age 46 years, female, “parent/carer” group]	
	Tone	16		*It was worded very factually and formally which is good when you’re looking at facts, but I think to make it more approachable for people if you used more informal language but obviously still making it accurate information, I think that would make it seem easier to process for people.* [age 15 years, female, “help for me” group]	
	Tone	17		*If somebody clicks something like you know the bit where it’s like, “seeking help for myself,” you’re like, “I’m really glad that you’ve reached out to us.” Or if somebody clicks a particular button, that’s so valid. Here’s some resources.* [age 17 years, gender diverse, “help for me” group]	
	Tone	18		*Even if you had a line that said at the start, “it is not uncommon to feel alone in this.” Because that’s probably how support people are feeling, however help is available to reduce your isolation and your fears or whatever like that.* [age 54 years, female, “parent/carer” group]	
**Key theme 3: Flow**					
	Navigation	19		*I like the idea of just buttons. I think it’s just easier to use. Especially because I feel like it’s hard to describe it sometimes – what you’re feeling and what you want to know about, so having the buttons just makes it easier because you can just look at what you want.* [age 17 years, male, “help for me” group]	
	Navigation	20		*I do like the idea of having obviously more things come up as you’re reading one because then it feels a lot more flowy, as opposed to seeing something going back. It feels like you’re having more of a conversation.* [age 17 years, transgender male, “help for me” group]	
	Navigation	21		*I think a lot of people going on it might not know specifically what they’re looking for, so having to type it all out, you might not even know what you want. So I think the buttons are good because it’s all laid out there in front of you instead of looking for anything.* [age 15 years, female, “help for me” group]	
	Navigation	22		*I think the buttons are okay, but I wonder if they can be ordered differently. I guess if you’re going into something like this, you’re probably looking for more immediate kind of answers and help so as a carer, you’re probably looking at the “what should I look out for?”* [age 46 years, female, “parent/carer” group]	
	Navigation	23		*I found the navigating really easy and the way that it told you what buttons you’d pushed in the history. That’s really good.* [age 53 years, female, “parent/carer” group]	
	Purpose	24		*I think we’re just in such a hurry for information so I was looking for a survey that would tell whether my kid is okay or not. I just want to know if he’s okay.* [age 55 years, female, “parent/carer” group]	
	Purpose	25		*I would be giving them step-by-step instructions. This will empower you when you go to the doctor [with your child].* [age 54 years, female, “parent/carer” group]	
	Purpose	26		*To have something that people can use 24/7 is really important when they can’t access any other help or just to get ideas of where to go.* [age 54 years, female, “parent/carer” group]	

### Key Theme 1: Chatbot Character and Design

#### Diverse Appeal

Overall, both groups of participants were satisfied with the appearance of KIT, the character, and reported that it would appeal to a wide audience of users. Favorable comments were made regarding the nongender specific design of KIT (quotes 1, 2, and 5) as participants reported this would appeal to users across the gender spectrum. This was noted to be important given the high correlation of eating disorders and body image concerns among gender diverse people [30].

Both groups of participants found the nonhuman design of KIT to be important as it felt safe and unlikely to trigger body image concerns (quotes 3 and 4). The participants in the parent/carer role provided particularly favorable comments regarding the nonhuman design due to multiple concerns raised that young people would compare their appearance to the character. It was expressed that “the less body parts the better” for the chatbot character. There was some discussion regarding the age appropriateness of KIT’s character, as both groups reported that it was slightly more targeted toward youth, but overall did not appear too “childish.” However, the parents/carers reported that it was more important for the character to appeal to young people and users rather than solely appealing to older users.

The young people in the “help for me” focus groups commented on the importance of including the pictorial character of KIT within the conversation text so it still seemed like they were conversing with “someone,” even though they knew this was a nonhuman chatbot. Interspersing images of KIT, the character, within the lengthy written information increased engagement and useability.

#### Visual Appeal

Both groups were positive regarding the design and style of KIT’s character and described KIT as “wholesome,” “engaging,” “resourceful,” and “nonjudgemental” (quotes 6-10). KIT’s expressions were reported by both groups to appeal to young people in particular and were perceived as engaging and approachable. Overall, the two groups of participants approved of the color scheme, with the majority of young people perceiving blues and greens as calming colors. When asked to describe their first impressions of KIT, the opinions of both groups were overwhelmingly positive, including “knowledgeable,” “inviting,” “approachable,” “kind,” “fun,” “professional,” and “cheerful.” These impressions centered around the idea of KIT as a character who could provide resources and high-quality information, while also appearing friendly and engaging (quote 6).

In addition to the visual design of the character, parent/carer and young participants commented upon the design of the webchat. Of importance to the parents/carers was the design, which was perceived as simple and thus promoting useability, as compared to other mental health resources that can appear “complicated” and “overwhelming.” The design of the webchat page was described as “clean and modern,” which was reported to be appealing and accessible for both groups of participants.

### Key Theme 2: Content Presentation

#### Brevity

The majority of participants in the two groups regarded the content of the webchat as positive and reported that this would be best presented in smaller sections (than was included in the prototype) to avoid overwhelming the user (quote 11). The preference for brevity was noted by both groups. It was expressed that individuals seeking this information may be feeling stressed and scared, and experiencing a sense of urgency; therefore, “short and sharp” content was reported to be more supportive.

Both groups of participants liked the use of emojis as dot points to enhance readability of the text (quote 13). In addition, the ideas of staggered messages, a “click for more” button and putting the content into multiple message bubbles (quote 12) were also raised to optimize the presentation of the written information within the chatbot. Some participants related this to receiving a long message from a friend and preferring multiple shorter messages instead. The young people and parents/carers reported that breaking up the content into “chunks” would feel more “conversational and interactive.” Participants from both groups appreciated the strategy of providing some information with additional resources available via external weblinks (quotes 14 and 15). This format was reported to be more inviting and accessible so users could explore the content at their own pace.

#### Tone

The interactivity of KIT was discussed in relation to the tone. Most participants from both groups reported that in addition to the factual information KIT presented, KIT could include supportive statements (eg, “I’m really glad that you’ve reached out to us” [quote 17]), which would provide a more conversational tone as well as normalize any concerns of the user (quote 17). Amongst the younger people in the “seeking help for myself” group, these participants preferred a more casual tone of conversation, while acknowledging it was necessary to present the information accurately (quote 16).

The parent/carer participants reported comments regarding the guided nature of the content, so they could feel reassured by the information, given their own experiences of feeling isolated, scared, and lost, as carers of young people with eating disorders (quote 18). Parents/carers reported that it was important not to “soften” the information, and alternatively present factual and realistic guidance, given the severity and risk of death associated with eating disorders [4]. All participants from both groups agreed that it was important for the content to be presented as factual and accessible, while balancing the supportive tone required.

### Key Theme 3: Flow

#### Navigation

The majority of participants in both groups reported that the button-based navigation was sufficient in navigating the chatbot rather than free text. The younger participants reported this was useful for people who might not know specifically what information or support they require (quotes 19 and 21). The buttons appealed to broader accessibility, with parent/carer and younger participants citing that the buttons would be beneficial for those with language difficulties. The buttons were reported to cover the relevant information, and the navigation was described as simple and straightforward to use (quote 23).

Some participants in both groups expressed that a free text option would be beneficial for more specific questions, but also described frustration at the current level of artificial intelligence technology available in other chatbots whose natural language processing was not sufficiently capable of understanding their typed queries. Generally, the parents/carers expressed a preference for having both button-directed navigation as well as the option to type in specific questions. These participants expressed that individuals using the “help for another” pathway would likely already be concerned about their loved one and would by this point have specific questions to which they would be seeking answers.

Regarding the flow of the chatbot conversation, some parents/carers and younger people suggested having “categories” where a user could flow through these suggested themes or having “next” buttons for relevant sections. Improving the flow of the conversation was considered important to enhance the interactivity of the experience. Some young people reported getting “decision fatigue” when “overwhelmed” with multiple buttons, and reported that merging the sections into categories would be helpful. The main feedback from parents/carers was to change the order of how information was presented in the buttons, as they reported that the immediate questions of “what do I look out for?” and “how can I help my child?” were the most important (quote 22). Both groups of participants appreciated seeing the history of the chat and the ability to scroll back and reread the conversation.

#### Purpose

One point of difference between the two groups of focus group participants was that the parent/carer group discussed the purpose and capabilities of the chatbot. This included queries around the proposed audience of the chatbot and how it might differ from other available resources. Some participants reported conversing with the chatbot with a specific question in mind, such as “Should I be worried about my child?” or “Where can I find a psychologist?” (quote 24). The researchers provided feedback regarding the ethical and practical limitations of the chatbot, with the purpose focused on providing psychoeducation and “in the moment” coping skills. It was apparent that the younger users were more familiar with the purpose and limitations of such technology, whereas the parents/carers presented different expectations regarding the specificity of their requests (quote 25). Both groups of participants praised the 24/7 availability of the chatbot as a major strength, particularly when other body image and eating disorder–focused supports are not available 24/7 (quote 26). In summary, the parents/carers reported that the chatbot would likely be a useful resource, and acknowledging the capabilities and limitations of the technology from the start of the conversation with KIT would be helpful to manage user expectations.

## Discussion

### Principal Findings

The aim of this study was to explore the preliminary acceptability and feasibility of a world-first chatbot designed to support people experiencing body image and eating concerns, and parents/carers. The qualitative results from the focus groups illustrated three main themes as follows: chatbot character and design, content presentation, and flow. Across these themes, both people seeking help for themselves and parents/carers generally provided positive feedback. The chatbot was praised for its clean design, ease of navigation, and engaging character. Both the groups approved of the gender nonconforming and nonhuman design of KIT, which was believed to improve the accessibility of the chatbot and help diverse users feel safe. Most participants approved of KIT’s facial expressions and found the character approachable, resourceful, and calming.

Most participants reported the content was appropriate, accessible, and helpful. To improve the chatbot, both audiences recommended presenting the amount of information differently, with a preference for “short and sharp” content. This preference for brevity was balanced by implementing a supportive tone and using opportunities to normalize concerns as well as provide information.

The participants seeking help for themselves preferred the navigational style using buttons, whereas the parents/carers requested the option to type specific queries as well as be guided through the content via buttons. This difference in preferences likely reflected the different mindsets these two audiences expressed. The young people preferred to be guided with the options laid out, whereas parents/carers often had specific queries and concerns. Overall, the participants expressed positive feedback regarding the body image chatbot, and specific recommendations were implemented in the development of KIT as per iterative agile methodology [31].

### Comparison With Prior Work

To our knowledge, this is the first chatbot designed to specifically target body image and eating concerns, with the majority of other digital mental health interventions focused on mood and anxiety disorders [24]. In addition, reports specifically addressing the development and design of chatbots in the field of mental health are rare [21,32]. However, our study found that users valued the chatbot’s useability, quality of content, and engagement, which was similar to previous research [33].

One area of divergence from the existing literature was related to the subtheme of brevity. A systematic review of mental health chatbots illustrated that most participants perceived conversations as too short or shallow [33]. However, in this study, participants reported a preference for shorter information. It is possible this difference reflects KIT’s strong focus on psychoeducational information, but nevertheless highlights the necessity of providing users with appropriate length responses for their needs.

Within the literature, the empathy of nonhuman agents has received much research attention, with evidence indicating the importance of the development of therapeutic bonds [20,34,35]. While there were recommendations to increase the interactivity of KIT within conversations, the use of a pictorial character within conversation text appeared to be a useful “shortcut” for building rapport with users.

Future research with larger sample sizes is required regarding the use and efficacy of KIT, which will provide user feedback and allow for further refinements of KIT’s content. After such evaluations, KIT can be more easily compared with other pre-existing and well-established mental health chatbots such as Woebot and Wysa [13,34,36]. With KIT’s focus on delivery of coping skills, particularly based on CBT, KIT is in line with chatbots like Woebot and Wysa, but has the unique focus of body image and eating issues compared to anxiety and depression [24].

### Strengths and Limitations

The main strength of this study was the diverse recruitment of participants for the focus groups, which strongly benefitted the co-design process of KIT. Both the parent/carer and young people groups included individuals from all regions of Australia owing to the online format. In the young people group, care was taken to include participants across the gender spectrum, including transgender and gender diverse individuals. This process included ensuring there was a mix of genders in each focus group. The majority of participants were female, which is reflective of the higher prevalence of eating disorders among women and girls. The study was limited by a smaller sample size. Furthermore, the sample was diverse in all but one demographic feature, which was ethnicity, with most participants identifying as Caucasian.

Given that participants were recruited through online advertising, bias is inherent in those who can access and respond to research advertisements. In addition, a small percentage of participants had previously been involved in preliminary online co-design forums for KIT, which may have influenced their opinions and feedback in the subsequent focus groups. Social desirability may have also influenced the feedback provided by participants, as the authors were conducting the focus groups. However, the authors strongly encouraged honest responses, and a range of positive, negative, and neutral comments were made by participants.

### Conclusions

Our study findings provide preliminary support for the acceptability and feasibility of KIT, the body image chatbot. The results showed overall positive feedback regarding the content, structure, and design of the body image chatbot, from both groups of participants in the “seeking help for myself” and “seeking help for another” pathways. The participants’ recommendations and feedback were used to refine KIT prior to public launch in order to improve the layout, content, and navigation of the chatbot. If proven effective, KIT would likely provide a valuable resource of psychoeducation and coping skills by meeting young people where they spend their time (online) and those who might not feel ready to follow traditional formats of seeking help. KIT may assist in filling the gaps in service delivery in terms of both prevention and intervention by supporting people and those concerned about them, with appealing and accessible evidence-based psychoeducation and skills.

## Abbreviations

ACT: acceptance commitment therapy
CBT: cognitive behavioral therapy
CBT-E: enhanced cognitive behavioral therapy
